# Leg‐fidgeting versus standing breaks during prolonged sitting: Impacts on blood pressure and heart rate in young women

**DOI:** 10.1113/EP093057

**Published:** 2025-09-11

**Authors:** Saja Alghamdi, Bethany Barone Gibbs, Ghareeb Omar Alshuwaier, Jamal M. Alzahrani, Abdullah Bandar Alansare

**Affiliations:** ^1^ Department of Exercise Physiology, College of Sport Sciences and Physical Activity King Saud University Riyadh Saudi Arabia; ^2^ Department of Epidemiology and Biostatistics West Virginia University School of Public Health Morgantown West Virginia USA

**Keywords:** cardiovascular health, female, interruption, physical activity, sedentary behaviour

## Abstract

The objective of this work was to examine whether leg‐fidgeting breaks during prolonged sitting could be a practical alternative to standing breaks in preventing blood pressure (BP) and heart rate (HR) impairments. Young women (*n* = 16; age = 21.9 ± 3.0 years; body mass index = 21.1 ± 4.9 kg/m^2^) completed three 3‐h prolonged sitting conditions in a random order, including: (1) uninterrupted prolonged sitting, (2) interrupted prolonged sitting every 20 min with standing for 5 min, and (3) interrupted prolonged sitting every 4 min with leg‐fidgeting for 1 min. Oscillometric brachial BP and HR were measured at baseline and after 1, 2 and 3 h of prolonged sitting. Generalized linear mixed models with random effects evaluated the effects of the three prolonged sitting conditions on BP and HR while controlling for baseline values. Effect sizes were estimated using Cohen's *d*. No significant differences were observed between the three prolonged sitting conditions for all BP variables (*P *> 0.05 for all). HR was significantly lower when prolonged sitting was interrupted with standing (β = −4.406 beats; *d *= 0.46; *P* = 0.009) or leg‐fidgeting (β = −3.802 beats; *d *= 0.46; *P* = 0.023) compared to the uninterrupted prolonged sitting condition. These findings suggest that leg‐fidgeting breaks during prolonged sitting may serve as a practical alternative to standing breaks in preventing some prolonged sitting‐induced cardiovascular impairments, particularly HR, in young women.

## Abstract (highlights)

- **What is the central question of this study?**
  Do leg‐fidgeting breaks serve as an effective substitute for standing breaks to confront cardiovascular dysregulations in young women?
- **What is the main finding and its importance?**
  While leg‐fidgeting breaks do not seem to effectively influence blood pressure, leg‐fidgeting breaks appeared to serve as a practical alternative to standing breaks in preventing prolonged sitting‐induced heart rate impairment in young women. Future randomized controlled trials of longer duration are essential to confirm sustained benefits (rather than acute) and among diverse populations.

## 1 INTRODUCTION

Sedentary behaviour (SB), such as prolonged sitting, is an established risk factor for cardiovascular disease (CVD) and CVD mortality (Gao et al., 2024). A recent analysis, which included 1,473,354 adults, estimated the risk of fatal and non‐fatal CVD to be 30% higher among those in high versus low SB categories (Onagbiye et al., 2024). It was also projected that high SB may contribute to more than 10% of fatal and non‐fatal CVD (Onagbiye et al., 2024). Despite the general declines in cardiovascular mortality over the past decades (Joseph et al., 2025; O'Flaherty et al., 2013), both CVD and SB remain alarmingly prevalent and are increasing worldwide (Bauman et al., 2018; Joseph et al., 2025). In response to this health challenge, global SB policies and recommendations are evolving to mitigate cardiovascular complications linked to SB (Alfawaz et al., 2021; DiPietro et al., 2020; Katzmarzyk et al., 2019).

The World Health Organization and others recommend that adults worldwide reduce overall time spent in SB and, importantly, break up prolonged sitting with light physical activity (PA) to promote cardiovascular and overall health (Alfawaz et al., 2021; Bull et al., 2020; Katzmarzyk et al., 2019). In agreement with these recommendations, systematic reviews of experimental studies suggest that interrupting prolonged sitting with walking, simple resistance PA, or standing improves cardiovascular health measures, including blood pressure (BP) and heart rate (HR) (Adams et al., 2024; da Silva et al., 2022; Paterson et al., 2022). However, these light PA strategies may not always be feasible under certain conditions, such as during commuting, travelling, or attending meetings. Moreover, emerging evidence indicates that standing breaks, especially if static and for long periods, may have adverse effects such as reduced circulation, blood pooling in the lower extremities and varicose veins, and increased vascular stiffness (Barone Gibbs et al., 2024; Messing & Dautel, 2025). Hence, testing practical SB breaks could help to mitigate CVD and CVD mortality from excessive, prolonged SB.

Recently, leg‐fidgeting, which is rapid and repetitive leg movements while seated, has arisen as a potentially effective light PA strategy that may prevent some prolonged sitting‐induced cardiovascular impairments. For example, popliteal artery blood flow, shear rate and flow‐mediated dilatation were augmented when prolonged sitting was interrupted with leg‐fidgeting compared to uninterrupted prolonged sitting in predominantly young male participants (Morishima et al., 2016). Likewise, breaking up prolonged sitting with leg‐fidgeting increased blood flow and velocity in the superficial femoral artery and reduced venous pooling in the lower limbs compared to uninterrupted prolonged sitting in young men (Fryer et al., 2023). However, leg‐fidgeting breaks during prolonged sitting did not influence BP or HR in young men (Fryer et al., 2023). Given that earlier research revealed greater cardiovascular benefits in women than men when interrupting prolonged sitting with light PA (Wheeler et al., 2019), young women may have better cardiovascular responses (e.g. BP or HR) to leg‐fidgeting breaks during prolonged sitting. This is an important research gap as the strategy of interrupting prolonged sitting with fidgeting breaks may be more feasible and an alternative to standing breaks for improving BP and HR in women.

Therefore, this study examined whether leg‐fidgeting breaks during prolonged sitting could be an effective alternative to standing breaks in reducing BP and HR among young women. It was hypothesized that leg‐fidgeting breaks during prolonged sitting would lower BP and HR to a comparable extent as standing breaks.

## 2 METHODS

### 2.1 Ethical approval

A written informed consent was obtained from all involved participants. The study conformed to the standards set by the *Declaration of Helsinki*, except for registration in a database. The protocol of this study was reviewed and approved by the Institutional Review Board at King Saud University (No. 23/0097/IRB‐A; May 29th, 2023).

### 2.2 Participants recruitment

A convenience sampling approach was employed to recruit the study's participants. To avoid potential health‐related confounding and improve the internal validity of the results, the following eligibility criteria were utilized: (1) non‐pregnant female, (2) non‐smoker, (3) between 18 and 45 years old, (4) resting systolic BP ≤ 140 mmHg and diastolic BP ≤ 80 mmHg (McCormack et al., 2019), (5) free of CVD or diabetes, and (6) not taking CVD or diabetes medications. The study's advertisement was distributed through the official King Saud University emails and social media platforms (e.g., WhatsApp, X). All experimental procedures were conducted in the Exercise Physiology Laboratory at King Saud University.

### 2.3 Experimental design

**FIGURE 1:**
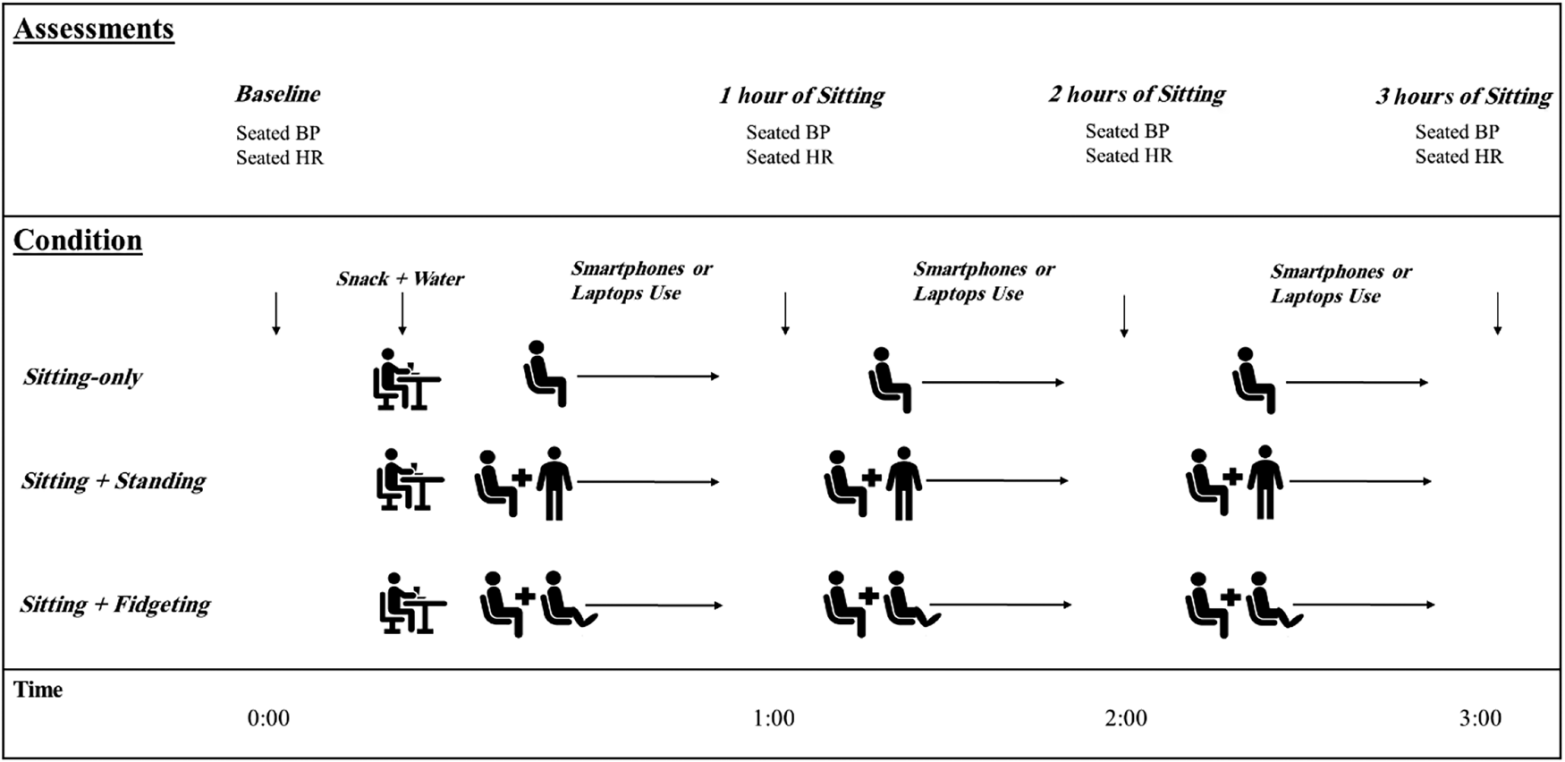
Experimental conditions. All participants arrived at the laboratory between 07.00 and 10.00 h for the three prolonged sitting conditions. Seated BP and HR were assessed, respectively, at four time points (baseline, 1, 2 and 3 h of sitting). During each prolonged sitting condition, baseline assessments were completed, a snack and water were consumed, and then the prolonged sitting bout started. Further assessments were completed after each hour of prolonged sitting. Abbreviations: BP, blood pressure; HR, heart rate.

This study utilized a randomized crossover experimental design. Participants completed three 3‐h prolonged sitting conditions (sitting‐only, sitting + standing, and sitting + leg‐fidgeting) in a random order with a 1‐week interval between visits. The participants were instructed to abstain from food for 10 h, and exercise and caffeine for 24 h prior to each visit. Upon arriving at the laboratory between 07.00 and 10.00 h, abstention as instructed was verbally confirmed, and a written informed consent was obtained. Then, baseline assessments, including BP eligibility measurements, were performed. Eligible participants were randomly assigned to perform 3 h of uninterrupted prolonged sitting (sitting‐only), 3 h of prolonged sitting with interruptions every 20 min with standing for 5 min (sitting + standing), or 3 h of prolonged sitting with interruptions every 4 min with leg‐fidgeting for 1 min (sitting + leg‐fidgeting; described in detail below) (Figure 1). During each condition, BP and HR assessments were completed at four time points (i.e. baseline and after 1, 2 and 3 h of prolonged sitting). A member of the research team was present during the entire three prolonged sitting conditions to ensure that participants remained seated throughout, except for standing or fidgeting activities following the study's protocols.

### 2.4 Randomization

The order of the experimental conditions for each participant was determined using a simple randomization method. One of the research team wrote sitting‐only, sitting + standing or sitting + leg‐fidgeting separately on three pieces of paper and folded them to hide the writing. Then, each participant selected the folded papers consecutively to respectively determine the first, second and third conditions. The allocation sequence was concealed from the participants after they selected the order, and they were informed of the assigned condition at the beginning of each experimental session.

### 2.5 Prolonged sitting conditions

Each participant completed three 3‐h experimental conditions: sitting‐only, sitting + standing and sitting + leg‐fidgeting. Prior to beginning each experimental condition, the participants were encouraged to empty their bladders. For the sitting‐only condition, participants sat continuously in a chair with the soles of both feet flat on the floor, the angle of the knee and hip joints at 90°, and their backs supported. Participants were allowed to use their smartphones or laptops. For the sitting + standing condition, participants performed the same procedures as the sitting‐only condition except that they were instructed to stand (assume a stationary upright posture) for 5 consecutive minutes after every 20 min of prolonged sitting. For the sitting + leg‐fidgeting condition, the participants also performed the same procedure as the sitting‐only condition, except that they were instructed to fidget their legs in sync with a metronome set at 250 taps/minute for 1 min, after every 4 min of prolonged sitting (Fryer et al., 2022). The leg‐fidgeting movement was performed as follows: keeping the toes of both feet on the floor while raising heels off the floor and then lowering them back down. Research personnel informed the participants to begin and stop leg‐fidgeting throughout the entire condition. To ensure that the participants adhered to their prolonged sitting protocols, a member of the research team observed the participants throughout each condition.

### 2.6 Snack and water

Before starting the prolonged sitting conditions, a standardized meal replacement drink (Ensure Plus, Ensure, Abbott Park, IL, USA; 5 kcal/kg body weight) consisting of 31% carbohydrate, 22.3% protein and 14% fat was provided to the participants, who were instructed to drink it within 5 min (Kerr et al., 2017). This standardized drink was distributed to mitigate the influence of hunger on the study's measurements. The participants were also offered 200 mL of water during each prolonged sitting condition that could be consumed ad libitum.

### 2.7 Measurements

#### 2.7.1 Body mass index

Each participant self‐reported their age. Body height and weight were measured in duplicate using a wall‐mounted stadiometer (Perspective Enterprises, Portage, MI, USA) and a digital scale (WB 110A, Tanita, Tokyo, Japan), respectively. The average of these measurements was utilized to calculate body mass index (BMI) as follows: BMI = body weight (kg)/body height (m^2^).

#### 2.7.2 Blood pressure and heart rate

A validated oscillometric device (e‐sphyg 2, Model: 9002, American Diagnostic Corporation, Hauppague, NY, USA) was utilized to measure BP (mmHg) and HR (beats/min). Participants’ BP and HR were measured after sitting quietly for 5 min with both feet flat on the floor, the angle of their knee and hip joints at 90°, arms supported at heart level, and backs supported in a chair (Whelton et al., 2018). They were instructed not to talk or move while resting and during the measurements. Two consecutive measurements were performed, separated by a 1‐min interval. The averages of these two measurements were utilized to determine systolic BP (SBP), diastolic BP (DBP), mean arterial pressure (MAP), pulse pressure (PP) and HR. All BP and HR measurement procedures were followed for eligibility, baseline and during the experimental condition after 1, 2 and 3 h.

#### 2.7.3 Sample size

The G Power software (G* Power Version 3.1.9.4) was utilized to calculate the sample size needed for this study. Assuming a within‐subject correlation of 0.7, a type I error rate of 0.05 and 80% power, 15 participants were needed to observe a standardized effect size of 0.3 (small to medium) within participants across the three experimental conditions. Sixteen participants were enrolled to account for potential missing data or withdrawals.

#### 2.7.4 Statistical analyses

Participants’ characteristics were reported using means and standard deviation (SD). To address the study's hypothesis, generalized linear mixed (GLM) models with random effects evaluated the effects of the conditions (sitting‐only, sitting + standing, and sitting + leg‐fidgeting) on BP and HR while controlling for baseline values (Kenward & Roger, 2010). In these GLM models, participant ID was specified as the random effect to account for within‐individual correlations across the conditions. The condition (sitting‐only vs. sitting + standing or sitting + leg‐fidgeting) was entered as the fixed effect, and baseline values were included as covariates to account for baseline differences. Each model was constructed to estimate two β coefficients and provided *P*‐values for between‐condition differences as follows: (1) the effect of sitting + standing versus sitting‐only and (2) the effect of sitting + leg‐fidgeting versus sitting‐only. Cohen's *d* was calculated as β/standard deviation of baseline values to estimate the effect size and clinical meaningfulness of the effects observed. These were classified using conventional thresholds (Cohen, 2013) of large if *d* = 0.8, medium if *d* = 0.5, or small if *d* = 0.2. All analyses were performed using SPSS Statistics software (Version: 28.0.0.0.190; IBM Corp., Armonk, NY, USA). The significance level for all analyses was set at *P* < 0.05.

## 3 RESULTS

Table 1 displays the general characteristics of the participants, who were young women with generally healthy resting BP, HR and BMI.

**TABLE 1:** General characteristics of the participants (*n* = 16).

Variable	Mean ± SD
Age (years)	21.9 ± 3.0
Height (cm)	161.3 ± 6.5
Weight (kg)	54.9 ± 12.9
BMI (kg/m^2^) Underweight (*n* (%)) Normal weight (*n* (%)) Overweight (*n* (%)) Obese (*n* (%))	21.1 ± 4.9 4 (25%) 8 (50%) 3 (18.6%) 1 (6.4%)
SBP (mmHg)	112.3 ± 9.1
DBP (mmHg)	69.6 ± 7.8
MAP (mmHg)	82.0 ± 14.0
PP (mmHg)	41.8 ± 10.1
HR (beats/min)	81.2 ± 9.3

Abbreviations: DBP, diastolic blood pressure; HR, heart rate; MAP, mean arterial pressure; PP, pulse pressure; SBP, systolic blood pressure.

**FIGURE 2:**
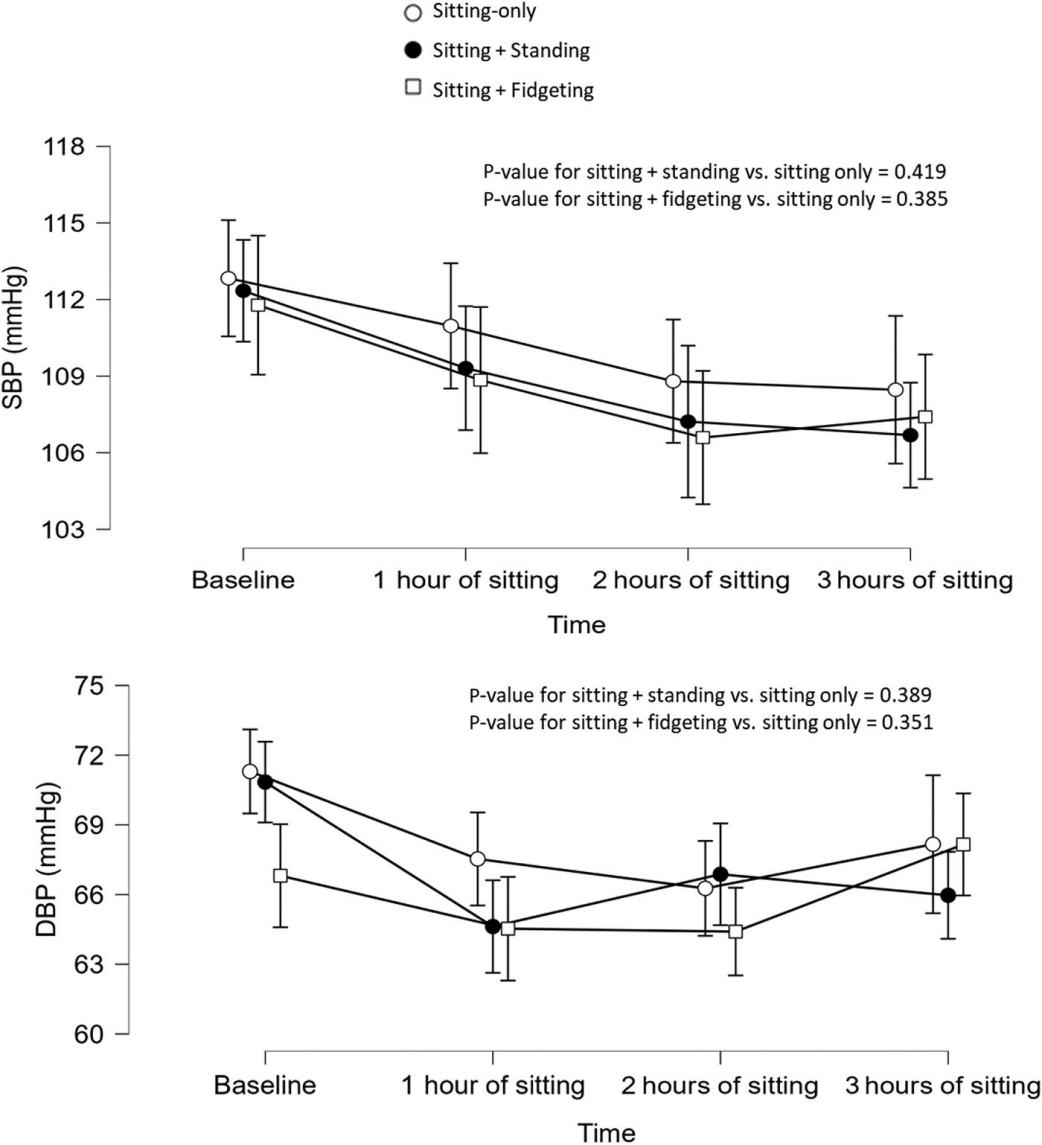
The effects of interrupting prolonged sitting with standing or leg‐fidgeting on systolic and diastolic blood pressure in women (*n* = 16). All models were adjusted for baseline values of the outcome. DBP, diastolic blood pressure; SBP, systolic blood pressure.

Figure 2 and Supporting information Table S1 show SBP and DBP across the sitting‐only, sitting + standing and sitting + leg‐fidgeting conditions. No significant differences were observed between the sitting + standing or sitting + leg‐fidgeting condition compared to the sitting‐only condition for SBP (β = −1.7 and −1.8 mmHg, respectively; *P* > 0.05) or DBP (β = −1.5 and −1.6 mmHg, respectively; *P* > 0.05). The sizes of these differences were small to medium (*d* ranged between 0.16 and 0.22). Notably, most of these BP variables tended to decrease over time, even in the control group.

**FIGURE 3:**
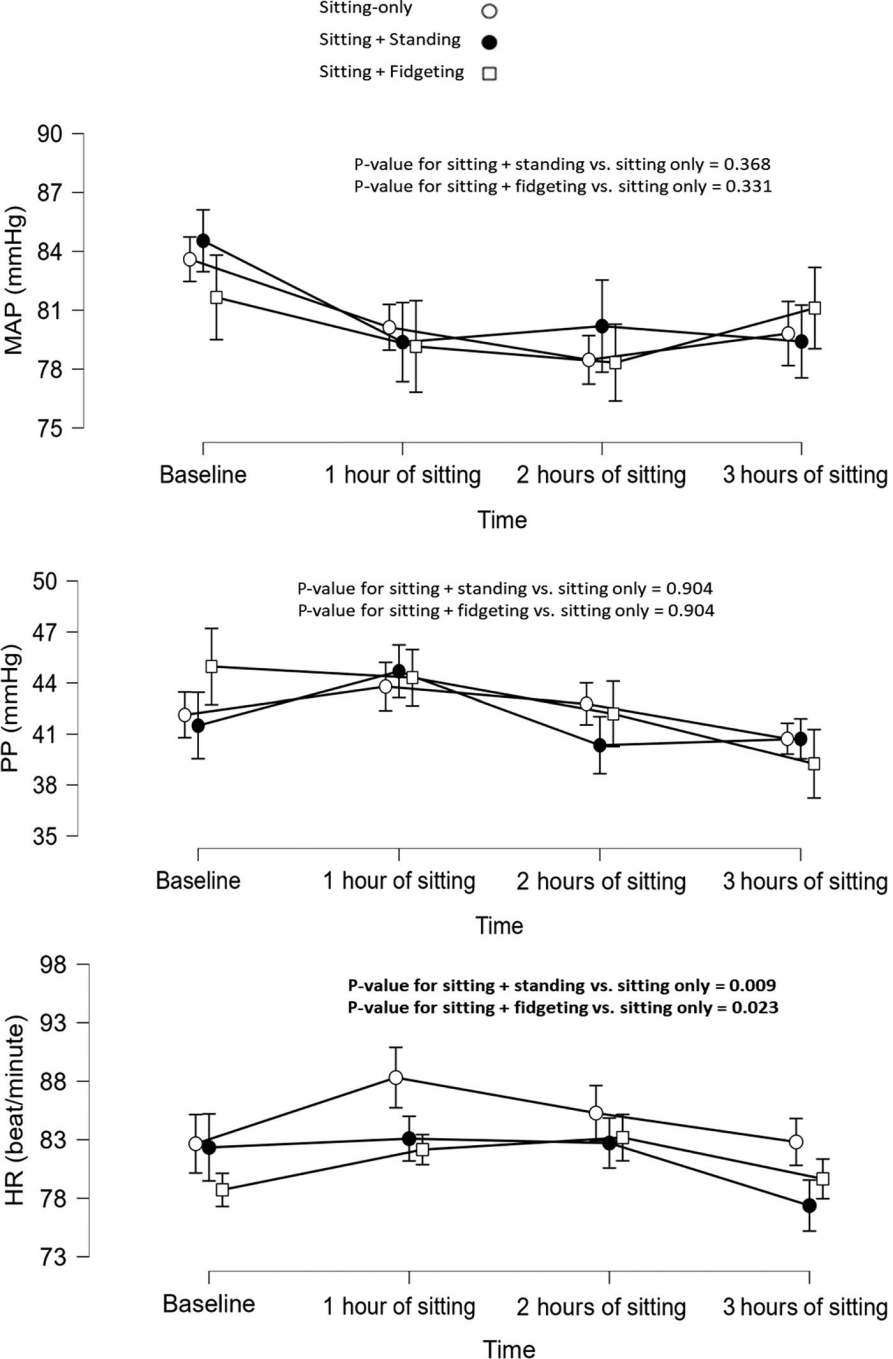
The effects of standing or leg‐fidgeting breaks during prolonged sitting on mean arterial pressure, pulse pressure and heart rate in women (*n* = 16). All models were adjusted for baseline values. HR, heart rate; MAP, mean arterial pressure; PP, pulse pressure.

Figure 3 and Supporting information Table S1 display MAP, PP and HR during the sitting‐only, sitting + standing, and sitting + leg‐fidgeting conditions. No significant differences were detected between the sitting + standing or sitting + leg‐fidgeting conditions compared to the sitting‐only condition for MAP (β = −1.6 and −1.7 mmHg, respectively; *P* > 0.05) or PP (β = −0.2 and −0.2 mmHg, respectively; *P* > 0.05). The sizes of these differences were also small to medium (*d* ranged between 0.02 and 0.42). However, HR was significantly lower in the sitting + standing or sitting + leg‐fidgeting conditions compared to the sitting‐only condition (β = −4.4 and −3.8 beats, respectively; *P* < 0.05). In addition, the sizes of these differences were small to medium (*d *= 0.46 and 0.36, respectively).

## 4 DISCUSSION

The present study uniquely explored the effectiveness of leg‐fidgeting breaks during prolonged sitting as a practical alternative to standing breaks in reducing peripheral BP and HR among young women. Neither standing nor leg‐fidgeting breaks during prolonged sitting elicited significant differences in BP variables compared to sitting only. In contrast, HR was significantly and comparably lower when prolonged sitting was interrupted with leg‐fidgeting or standing in young women. These findings suggest that leg‐fidgeting breaks during prolonged sitting may serve as a practical alternative to standing breaks in preventing some prolonged sitting‐induced cardiovascular impairments in young women.

### 4.1 Strengths and limitations

The study has strengths that are worth mentioning. First, this investigation used a randomized crossover design to control for between‐participant variability, improving the precision of measuring treatment effects (Lim & In, 2021). Though this design has the risk of introducing carryover effects, the washout period minimizes this risk. Furthermore, earlier research separately examined the influence of leg‐fidgeting or standing breaks during prolonged sitting on cardiovascular measures while enrolling male‐only or majority male samples (Barone Gibbs et al., 2017; Fryer et al., 2023; Peddie et al., 2021). This constraint may limit the generalizability of the findings, particularly given mixed evidence regarding sex differences in cardiovascular responses to prolonged sitting, although findings in the literature are inconclusive (O'Brien et al., 2019; Vranish et al., 2017). As such, the present study overcame this limitation by including a female‐only sample and concurrently assessing the cardiovascular responses to leg‐fidgeting or standing breaks during prolonged sitting.

Still, some constraints existed in this study and should be considered when interpreting the current findings. Although the sample size was calculated to detect a standardized effect size of 0.3, it was limited to detecting smaller but potentially important influences on cardiovascular measures, particularly on BP, which appeared to show greater reduction in both interventions compared to the control condition, but not in a statistically significant way. Most of the BP variables tended to decrease over time, even in the control condition, which contrasts with previous evidence suggesting uninterrupted prolonged sitting raises BP (Adams et al., 2024). These inconsistencies may reflect inter‐individual variability (Teixeira & Millar, 2025), which limits the potential for detecting protective effects from the interventions in the current study. Moreover, the homogeneous sample (i.e. young female‐only), which benefits internal validity, also limits external validity (Jager et al., 2017).

The stage of the menstrual cycle or the use of oral contraceptives also was not controlled for in the current study, as it is not typically included in the current recommended practice for the measurement of BP or HR (Laborde et al., 2017; Mahe et al., 2017). Nonetheless, emerging evidence from prolonged sitting research suggests that vascular haemodynamics may be different between naturally menstruating women and those using oral contraceptive pills (O'Brien et al., 2020). This could have led to non‐differential variability in our findings, which would limit precision. Additionally, the two BP measurements were averaged without a similarity threshold or additional measurements, which might have increased variability and further reduced the precision. Finally, a standardized meal replacement drink was provided to increase external validity and reduce potential impacts of hunger or fasting on BP and HR, which, though comparable across conditions, could have influenced the responses. Future prolonged sitting studies in women should consider including a larger sample size, exploring inter‐individual variability in cardiovascular responses, accounting for the stage of the menstrual cycle or the use of oral contraceptives, and examining the influence of food intake.

### 4.2 Effects of standing or leg‐fidgeting breaks during prolonged sitting on BP

A recent systematic review and meta‐analysis demonstrated that interrupting prolonged sitting with standing, aerobic or simple resistance PA only decreased peripheral SBP and DBP by 0.24 mmHg/h in predominantly healthy adults (Adams et al., 2024); however, removing an outlier study (with two trials involving diabetic patients) resulted in the loss of the significant influence observed. Furthermore, the only existing study that examined the effects of leg‐fidgeting breaks during prolonged sitting on BP showed no significant alterations in peripheral or central BP in healthy young males (Fryer et al., 2023). The present study reinforces these findings and confirms no changes in brachial BP variables in healthy young females when interrupting prolonged sitting with standing or leg‐fidgeting.

A wealth of emerging evidence indicates that PA produces stronger BP reductions in patients with lifestyle‐related diseases compared to their healthy counterparts (Suematsu et al., 2025), and these diminutions may more strongly manifest with higher intensity PA (Marcal et al., 2021). In parallel with this, most BP variables in the current study tended to decrease over time, even in the control condition, a finding not aligned with earlier research (Adams et al., 2024). This discrepancy may reflect inter‐individual differences in BP responses to physiological stressors (Teixeira & Millar, 2025). Collectively, although it was speculated that neither standing nor leg‐fidgeting breaks during prolonged sitting could reach the optimal PA intensity that promotes BP reductions in young adults without known health conditions, the inter‐individual variability may have contributed to these null responses. Therefore, there is a need for research to rigorously investigate leg‐fidgeting versus standing breaks during prolonged sitting on BP variables in patients with lifestyle‐related diseases and explore inter‐individual differences in BP responses to prolonged sitting with and without interruptions.

### 4.3 Effects of standing or leg‐fidgeting breaks during prolonged sitting on HR

A prior systematic review and meta‐analysis found that interrupting prolonged sitting with standing did not significantly influence HR in adults with diverse health profiles (Bates et al., 2021); notably, the samples of the included trials in these analyses mainly consisted of male‐only or mixed‐sex individuals. In line with this finding, the only known study that assessed the effects of leg‐fidgeting breaks during prolonged sitting on HR also observed no significant HR alternations in young healthy men (Fryer et al., 2023). Diverging from these observations, the current study revealed that interrupting prolonged sitting with standing or leg‐fidgeting breaks significantly decreased HR (−4.406 and −3.802 beats, respectively) in young healthy women. These discrepancies may be explained by variations in the length of prolonged sitting (e.g. ≤3 h vs. ≥4 h) (Bates et al., 2021), types of meals provided during prolonged sitting (e.g., a low‐fat meal replacement versus a high‐fat meal) (Fryer et al., 2023) and participant sex differences (e.g., female only, male only or mixed sex samples) (Bates et al., 2021; Fryer et al., 2023). Prior reports have shown that cardiovascular responses vary significantly across different meals (Millis et al., 2009), prolonged sitting periods (Bates et al., 2021) and sex (Vranish et al., 2017). Hence, further investigations that consider these methodological concerns are warranted to achieve a more thorough understanding of cardiovascular responses to interrupted prolonged sitting in adults.

### 4.4 Possible mechanisms

Earlier SB research proposed potential mechanisms linking prolonged sitting with cardiovascular impairments, and how PA breaks may combat these disruptions (Pekas et al., 2023; Stoner et al., 2021). To illustrate, uninterrupted prolonged sitting may acutely disturb cardiovascular functions by diminishing the muscular pump, increasing blood pooling in the lower extremities, and waning shear stress and nitric oxide bioavailability (Pekas et al., 2023; Stoner et al., 2021); these changes lead to decreased preload and cardiac output. To compensate, the cardio‐autonomic centre in the brainstem persistently increases its neural activity and augments BP and HR. On the contrary, PA breaks during prolonged sitting may reverse these mechanisms by activating the muscular pump, which precludes blood pooling in the lower extremities, promotes shear stress and nitric oxide bioavailability, and boosts preload and cardiac output (Pekas et al., 2023; Stoner et al., 2021). This reversal mechanism ultimately decreases BP and HR. This hypothesis is supported by the current findings, where HR significantly decreased when prolonged sitting was interrupted with standing or leg‐fidgeting, potentially reflecting improvements in preload and cardiac output. Yet, the unchanged BP may indicate that standing or leg‐fidgeting breaks during prolonged sitting may not be adequate to enhance shear stress, nitric oxide bioavailability, and, thus, BP.

### 4.5 Clinical implications

Acknowledging recent evidence indicating that standing breaks, especially prolonged static standing, may deleteriously affect cardiovascular health (Barone Gibbs et al., 2024; Messing & Dautel, 2025), exploring effective alternative strategies is of clinical significance to prevent adverse cardiovascular impacts resulting from prolonged sitting. The present study provides initial evidence for a potentially practical alternative to standing breaks, namely, leg‐fidgeting breaks. The findings suggest that interrupting prolonged sitting with leg‐fidgeting can have protective and promotional cardiac effects. These beneficial influences are comparable to those observed when prolonged sitting is interrupted with standing. Therefore, these observations encourage the integration of leg‐fidgeting breaks into the clinical recommendations and lifestyle interventions aimed at averting detrimental cardiovascular impacts induced by prolonged sitting. These endeavours may be better suited in conditions where posture transitioning is not feasible, such as during office work, meetings, or flights. Moreover, this SB reduction strategy may have greater applicability in patients with high, persistent resting HR (Heuer et al., 2024; Lin et al., 2025). Despite this, future long‐term randomized controlled trials that include diverse participants' characteristics (e.g. different age groups, health status and PA status) and measure central haemodynamics, as well as physiological changes specific to the lower limbs, are essential to confirm sustained benefits (rather than acute) and among diverse populations.

### 4.6 Conclusion

In short, this study assessed whether leg‐fidgeting breaks during prolonged sitting could be considered as a practical alternative to standing breaks in preventing cardiovascular dysregulations in young women. Although BP was not lower with either prolonged sitting interruption strategy, HR was attenuated to a comparable extent when prolonged sitting was interrupted with either standing or leg‐fidgeting. These encouraging findings suggest the potential effectiveness of leg‐fidgeting breaks during prolonged sitting as another option to avert some prolonged sitting‐induced cardiovascular impairments.

## AUTHOR CONTRIBUTIONS

Conceptualization, Saja Alghamdi and Abdullah Bandar Alansare; methodology, Saja Alghamdi and Abdullah Bandar Alansare; formal analysis, Abdullah Bandar Alansare; investigation, Saja Alghamdi, Bethany Barone Gibbs, Ghareeb Omar Alshuwaier, Jamal M. Alzahrani, and Abdullah Bandar Alansare; interpretation, Saja Alghamdi, Bethany Barone Gibbs, Ghareeb Omar Alshuwaier, Jamal M. Alzahrani, and Abdullah Bandar Alansare; data curation, Abdullah Bandar Alansare; writing—original draft preparation, Saja Alghamdi, Bethany Barone Gibbs, Ghareeb Omar Alshuwaier, Jamal M. Alzahrani, and Abdullah Bandar Alansare; writing—review and editing, Saja Alghamdi, Bethany Barone Gibbs, Ghareeb Omar Alshuwaier, Jamal M. Alzahrani, and Abdullah Bandar Alansare. All authors have read and approved the final version of this manuscript and agree to be accountable for all aspects of the work in ensuring that questions related to the accuracy or integrity of any part of the work are appropriately investigated and resolved. All persons designated as authors qualify for authorship, and all those who qualify for authorship are listed.

## FUNDING INFORMATION

This research received no external funding.

## CONFLICT OF INTEREST

The authors declare no conflicts of interest.

## Supporting information



Table S1. Effects of interrupting prolonged sitting with standing or fidgeting on cardiovascular measures in women (*n* = 16).

## Data Availability

The data are available upon request from the corresponding author.
