# Supplementary material for: Leg‐fidgeting versus standing breaks during prolonged sitting: Impacts on blood pressure and heart rate in young women
Source: Exp Physiol. 2025 Sep 11:10.1113/EP093057. Online ahead of print. doi: 10.1113/EP093057 (PMC13394803; doi:10.1113/EP093057)
Supplement: Supplementary file 1 — Table S1. Effects of interrupting prolonged sitting with standing or fidgeting on cardiovascular measures in women (n = 16). [file EPH-9999-0-s001.docx]

**Supplemental Table 1. Effects of interrupting prolonged sitting with standing or fidgeting on cardiovascular measures in women (n=16).**

| Outcome | Condition | 1-hour of sitting (mean ± SD) | 2-hour of sitting (mean ± SD) | 3-hour of sitting  (mean ± SD) | Sitting + Standing vs. Sitting-only | | Sitting + Fidgeting vs. Sitting-only | |
| --- | --- | --- | --- | --- | --- | --- | --- | --- |
|  |  |  |  |  | ß ± SE  (p-value) | *d* | ß ± SE  (p-value) | *d* |
| SBP (mmHg) | Sitting-only | 110.8 ± 9.2 | 109.3 ± 9.2 | 108.1 ± 10.9 | -1.672 ± 2.067 (0.419) | 0.21 | -1.797 ± 2.067 (0.385) | 0.16 |
|  | Sitting + Standing | 109.3 ± 9.7 | 107.2 ± 11.9 | 106.7 ± 8.2 |  |  |  |  |
|  | Sitting + Fidgeting | 108.8 ± 11.4 | 106.6 ± 10.5 | 107.4 ± 9.8 |  |  |  |  |
| DBP (mmHg) | Sitting-only | 67.2 ± 7.6 | 66.5 ± 7.7 | 68.1 ± 11.1 | -1.499 ± 1.741 (0.389) | 0.22 | -1.624 ± 1.741 (0.351) | 0.18 |
|  | Sitting + Standing | 64.6 ± 8.0 | 66.9 ± 8.8 | 66.0 ± 7.5 |  |  |  |  |
|  | Sitting + Fidgeting | 64.5 ± 8.9 | 64.4 ± 7.5 | 68.2 ± 8.8 |  |  |  |  |
| MAP (mmHg) | Sitting-only | 81.6 ± 7.3 | 80.6 ± 7.5 | 81.3 ± 10.8 | -1.556 ± 1.730 0.368)) | 0.25 | -1.681 ± 1.730 (0.331) | 0.20 |
|  | Sitting + Standing | 79.4 ± 8.1 | 80.2 ± 9.4 | 79.4 ± 7.4 |  |  |  |  |
|  | Sitting + Fidgeting | 79.2 ± 9.3 | 78.3 ± 7.8 | 81.1 ± 8.3 |  |  |  |  |
| PP (mmHg) | Sitting-only | 43.7 ± 7.8 | 42.8 ± 7.2 | 40.0 ± 5.2 | -0.172 ± 1.424 (0.904) | 0.02 | -0.172 ± 1.424 (0.904) | 0.02 |
|  | Sitting + Standing | 44.7 ± 6.2 | 40.3 ± 6.7 | 40.7 ± 4.7 |  |  |  |  |
|  | Sitting + Fidgeting | 44.3 ± 6.6 | 42.2 ± 7.7 | 39.3 ± 8.1 |  |  |  |  |
| SBPv (mmHg) | Sitting-only | 4.1 ± 3.6 | 3.4 ± 2.6 | 4.6 ± 2.8 | 0.499 ± 0.846 (0.556) | 0.09 | 1.207 ± 0.846 (0.154) | 0.23 |
|  | Sitting + Standing | 2.8 ± 2.6 | 7.1 ± 7.7 | 3.8 ± 2.6 |  |  |  |  |
|  | Sitting + Fidgeting | 4.8 ± 4.2 | 5.3 ± 4.0 | 5.6 ± 3.4 |  |  |  |  |
| DBPv (mmHg) | Sitting-only | 6.8 ± 4.4 | 5.4 ± 3.4 | 3.4 ± 2.6 | -1.449 ± 0.742 (0.051) | 0.33 | -0.824 ± 0.742 (0.267) | 0.21 |
|  | Sitting + Standing | 4.1 ± 3.1 | 3.4 ± 3.0 | 3.7 ± 3.3 |  |  |  |  |
|  | Sitting + Fidgeting | 2.7 ± 2.2 | 4.3 ± 3.8 | 6.1 ± 4.9 |  |  |  |  |
| HR (beats/minute) | Sitting-only | 88.3 ± 10.3 | 85.3 ± 9.4 | 82.8 ± 8.0 | **-4.406 ± 1.676 (0.009)** | **0.46** | **-3.802 ± 1.676 (0.023)** | **0.36** |
|  | Sitting + Standing | 83.1 ± 7.6 | 82.7 ± 8.5 | 77.4 ± 8.7 |  |  |  |  |
|  | Sitting + Fidgeting | 82.2 ± 5.1 | 83.2 ± 7.9 | 79.7 ± 6.8 |  |  |  |  |

ß; beta coefficient, *d*; Cohen's *d*, DBP; diastolic blood pressure, DBPv; diastolic blood pressure variability, HR; heart rate, MAP; mean arterial pressure, mmHg; millimeter of mercury, PP; pulse pressure, SBP; systolic blood pressure, SBPv; systolic blood pressure variability, SD; standard deviation, SE; standard error. All models were adjusted for baseline values of the outcome. Bold indicates significant association (p<.05).
